# PCSK9 Predicts Adverse Limb Outcomes After Endovascular Revascularization in Diabetic Chronic Limb-threatening Ischemia

**DOI:** 10.1055/a-2717-3194

**Published:** 2025-10-17

**Authors:** Federico Biscetti, Maria Margherita Rando, Maria Anna Nicolazzi, Giorgia Polito, Giovanni Pecorini, Flavia Angelini, Roberto Iezzi, Luis H. Eraso, Paul J. Dimuzio, Dario Pitocco, Massimo Massetti, Antonio Gasbarrini, Andrea Flex

**Affiliations:** 1 Cardiovascular Internal Medicine Unit, Fondazione Policlinico Universitario A. Gemelli IRCCS, Roma, Italy; 2 Department of Translational Medicine and Surgery, Università Cattolica del Sacro Cuore, Roma, Italy; 3 Radiology Unit, Fondazione Policlinico Universitario A. Gemelli, IRCCS, Roma, Italy; 4 Division of Vascular and Endovascular Surgery, Thomas Jefferson University, Philadelphia, Pennsylvania, United States; 5 Diabetology Unit, Fondazione Policlinico Universitario A. Gemelli, IRCCS, Roma, Italy; 6 Department of Cardiovascular Sciences, Fondazione Policlinico Universitario A. Gemelli IRCCS, Roma, Italy; 7 Department of Medical and Surgical Sciences, Fondazione Policlinico Universitario A. Gemelli IRCCS, Roma, Italy

**Keywords:** peripheral arterial disease (PAD), chronic limb-threatening ischemia (CLTI), proprotein convertase subtilisin/kexin type-9 (PCSK9)

## Abstract

**Background and Aims:**

Proprotein convertase subtilisin/kexin type-9 (PCSK9) plays a crucial role in pathophysiologic processes leading to limb and cardiovascular complications in diabetes, including cholesterol homeostasis, inflammation, and endothelial oxidative stress. This study examined the association between PCSK9 levels and major adverse limb events (MALEs) in patients with type 2 diabetes mellitus (T2DM) and chronic limb-threatening ischemia (CLTI) after endovascular revascularization.

**Methods:**

This prospective cohort study included 147 T2DM patients with peripheral artery disease undergoing endovascular revascularization for CLTI. Clinical assessments, including PCSK9 blood levels, were performed, and patients were followed for 12 months to monitor MALEs. Logistic regression and ROC curve analyses assessed the relationship between PCSK9 and MALEs.

**Results:**

During follow-up, 53 patients experienced MALEs. These patients were younger and had more severe peripheral artery disease. PCSK9 levels were significantly higher in those with MALEs (410.5 ng/mL) versus those without (360.6 ng/mL). ROC analysis showed that adding PCSK9 to cardiovascular risk factors improved MALE prediction. PCSK9 levels and Rutherford 4 category were independent risk factors for MALEs.

**Conclusion:**

Elevated PCSK9 levels are strongly associated with increased MALE risk in T2DM patients and may influence age of presentation and disease severity in CLTI. These findings highlight PCSK9 as a potential predictive biomarker and therapeutic target for vascular complications.

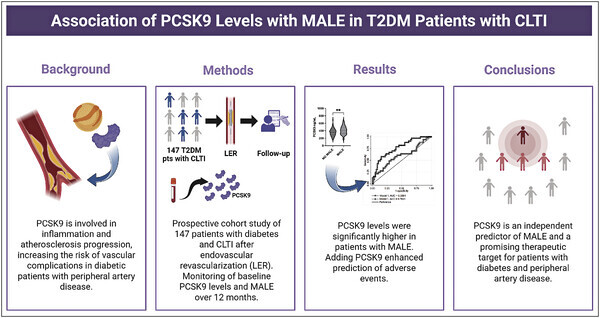

## Introduction

In patients with type 2 diabetes mellitus (T2DM), peripheral arterial disease (PAD) has distinctive phenotypic characteristics compared with non-diabetic individuals including a predisposition for infra-popliteal disease, a more severe level of disease, and younger age of presentation. 1 2 3 PAD is often associated with coronary artery disease (CAD) and cerebrovascular disease (CVD) in the setting of a polyvascular disease, affecting more than 200 million people worldwide, significantly impacting quality of life, and constituting a social and economic burden on the healthcare system. 4 5

Management strategies for PAD encompass lifestyle changes, pharmacotherapy, and, in cases of critical limb ischemia, revascularization procedures. 1 6 7 Revascularization strategies include endovascular and open surgical approaches, with endovascular procedure recently becoming an increasingly effective treatment option for improving prognosis and symptom relief for patients with PAD. 1

Post revascularization, maintaining rigorous risk modification therapy is crucial to avert complications, including the risk of restenosis and major adverse limb events (MALEs) such as acute limb ischemia, limb-threatening ischemia needing urgent revascularization, and major amputations. 6 8 Postoperative medical management aims to minimize the atherosclerotic and thrombotic burden 6 9 10 11 ; however, despite optimal treatment, many patients still experience adverse limb outcomes post revascularization. 10 This highlights the necessity for identifying biomarkers that can aid in risk stratification and represent targets to monitor response to novel therapeutic interventions beyond traditional risk factors.

Cardiovascular complications in patients with diabetes are associated with multiple mechanisms.

Specifically, our previous research has illustrated in patients with T2DM the significant association between inflammation biomarkers and the risk of MALEs and major adverse cardiac events (MACEs) post endovascular revascularization, 1 12 13 along with factors related to lipid metabolism and calcium homeostasis. 14 15 16 Cholesterol plays a key role in the formation and progression of atherosclerotic plaques. Among the molecules influencing atherosclerosis progression and connected with lipid metabolism, but not part of lipid particles, is the proprotein convertase subtilisin/kexin type 9 (PCSK9). The primary role of PCSK9 in atherogenesis involves degrading LDL receptors (LDLr), thereby elevating circulating LDL cholesterol (LDL-C) levels. 17 Beyond this, PCSK9 is implicated in various pleiotropic and pro-inflammatory mechanisms, which play a critical role in the development of atherosclerotic cardiovascular disease. 17 PCSK9 has direct and indirect pro-inflammatory effects mediated by interactions with oxidized lectin-like lipoprotein-1 (LOX-1) and other scavenger receptors (SRs). 17 The secretion of PCSK9 is not limited to hepatocytes but also occurs in endothelial cells, VSMCs, and macrophages, and is upregulated by factors like tumor necrosis factor (TNF)-α and lipopolysaccharide (LPS). 17 Moreover, PCSK9 can induce the secretion of pro-inflammatory cytokines from macrophages, liver cells, and a variety of other tissues, and it can regulate the expression of Toll-like receptor (TLR)-4 and the activation of nuclear factor kappa-light-chain-enhancer of activated B cells (NF-κB), indicating its involvement in inflammation, apoptosis, and autophagy. 17

The role of PCSK9 inhibitors is well established as an effective intervention for lowering lipids and reducing cardiovascular adverse events. 18 19 20 21 22 Multiple studies have shown that in patients with diabetes, higher PCSK9 levels are associated with indicators of cardiovascular risk, such as arterial stiffness or carotid intima–media thickness (IMT). 23 24 PCSK9 levels are also associated with the presence of CAD and its severity. 25

In a study comparing patients with and without PAD, those with PAD had higher levels of PCSK9. 26 Moreover, in patients with PAD, elevated PCSK9 levels have been linked to a significantly higher risk of the condition, an association that remains robust even after adjusting for various risk factors. 26

Given the multifaceted impact of PCSK9 on cardiovascular health, it is conceivable that it could serve as a biomarker for the risk of atherosclerotic disease progression and major cardiovascular events in individuals with PAD and offer a tool for tailoring patient-specific therapeutic strategies. Thus, this prospective study investigated the potential role of PCSK9 as a biomarker for the risk of MALE following endovascular revascularization in patients with T2DM and PAD experiencing chronic limb-threatening ischemia (CLTI).

## Materials and Methods

### Study Design

This is a prospective cohort study designed to explore the relationship between levels of circulating PCSK9 and the occurrence of MALEs in patients with T2DM and PAD experiencing CLTI and who have undergone endovascular revascularization. The research received approval from the Ethics Committee of the Fondazione Policlinico Universitario A. Gemelli IRCCS (approval number: ID 1990 13072/18), ensuring compliance with the Declaration of Helsinki's ethical guidelines. Participation in the study was contingent upon receiving informed consent from all enrolled patients.

### Study Population and Clinical Assessment

From October 23, 2019 to October 30, 2022, 147 patients with T2DM and PAD, requiring endovascular revascularization for CLTI, were enrolled at the Cardiovascular Internal Medicine Unit of Fondazione Policlinico Universitario A. Gemelli IRCCS in Rome, Italy. To reduce selection bias, consecutive patients who met the predefined inclusion and exclusion criteria were prospectively enrolled. All patients followed a standardized diagnostic and therapeutic pathway, and data were collected using uniform procedures.

Eligibility for the study was determined based on the following criteria: individuals older than 18 years with a diagnosis of T2DM for at least 1 year; an Ankle/Brachial Index (ABI) below 0.80; ultrasound color Doppler (US) confirmation of lower-extremity arterial stenosis exceeding 50%; classification of PAD as category 4 or 5 according to the Rutherford classification 27 ; presence of CLTI as already defined 12 ; and necessity of endovascular revascularization.

Participants were excluded if they were pregnant; had chronic kidney disease with an estimated glomerular filtration rate (eGFR) below 30 mL/min as per the Chronic Kidney Disease Epidemiology Collaboration (CKD-EPI) formula; had undergone lower-extremity surgical or endovascular revascularization in the preceding month; had active solid or hematological malignancies; had undergone organ or bone marrow transplantation; were expected to live less than 12 months; had acute infectious diseases at enrollment or within the preceding 2 weeks; had autoimmune diseases; had liver disease classified as Child–Pugh B or C; had known or suspected monogenic hereditary dyslipidemias; had acquired or congenital thrombocytopenia or thrombophilia; had contraindications to antiplatelet therapy; had known congenital bleeding disorders or acquired coagulopathies; had contraindications to endovascular revascularization; or if the revascularization procedure failed to address the targeted lesion.

All the subjects enrolled in the study underwent ultrasound (US) assessment. Furthermore, US was performed to confirm the presence of severe stenosis of the lower extremity in all the patients with an ABI of 1.40 or higher. Osteomyelitis suspicions were excluded through radiological imaging.

Collected data included age, sex, body mass index (BMI), duration of diabetes, smoking status, history of hypertension, hypercholesterolemia, CAD, CVD, ABI, Rutherford classification, and results from laboratory tests. Upon enrollment, patients were on single antiplatelet therapy, which was escalated to dual antiplatelet therapy (DAPT) for 1 month post revascularization. All patients received statins and/or ezetimibe as part of their lipid-lowering treatment, with no use of PCSK9 inhibitors. Post revascularization, lipid-lowering therapy adjustments aimed for an LDL-C target of less than 55 mg/dL, following the ESC/EAS Guidelines for dyslipidemia management. 28

### Lower-extremity Endovascular Revascularization and Follow-up

Revascularization of the lower extremities was performed through balloon angioplasty and/or the insertion of stents, in line with methodologies detailed in previous studies. 12 14 29 All patients underwent below-the-knee (BTK) endovascular revascularization. The procedure predominantly consisted of plain balloon angioplasty. Stent implantation was performed only when necessary, such as in the presence of flow-limiting dissection or significant elastic recoil after balloon angioplasty. In our cohort, only three patients (2.0%) underwent stent placement. The procedure was deemed successful when post-treatment arterial vessel stenosis was reduced to less than 30%. Adhering to the Society of Interventional Radiology's criteria, no complications associated with the endovascular procedures were observed. 30 Over a 12-month follow-up period, patients were monitored at intervals of 1, 3, 6, and 12 months following the revascularization to evaluate the occurrence of MALEs. MALEs were identified as instances of acute limb ischemia, major vascular amputations, and limb-threatening ischemia that required immediate revascularization.

### Blood Test and Biochemical Analysis

Laboratory tests were collected at baseline, before lower-extremity revascularization, in all the subjects enrolled in the study. Glycated hemoglobin (HbA1c), fasting glucose (FBG), total cholesterol, LDL-C, triglycerides, and creatinine were analyzed. eGFR was determined according to CKD-EPI formula. Serum was prepared by centrifugation of blood samples, which was stored at −80°C until assayed. Serum levels of PCSK9 were determined by commercially available ELISA kits (EH0251 from Fine Test, Wuhan, China) according to their protocol. The assay was performed at our institutional laboratory using research funds.

The precision of the measurements is demonstrated by the intra-assay and inter-assay coefficients of variation, recorded at 3.5 and 10.5%, respectively. The assay's sensitivity reached 0.625 ng/mL, calculated from the mean ± 3 standard deviations of the 0 standard. For each patient, serum levels were quantified twice, and the results averaged to ensure accuracy.

### Statistical Analysis

For the power analysis and sample size calculation, we assumed an effect size (Cohen's d) of 0.4, an α level of 0.05, and a target power of 0.8. Based on these parameters, the required sample size was calculated to be 150. Demographic and clinical characteristics are presented as means with standard deviations or medians with interquartile ranges (25th–75th percentiles) for continuous data, and as counts with percentages for categorical data.

To compare baseline characteristics of the study population, Student's *t* -test was used for comparing means of normally distributed continuous variables, the chi-square test was applied for assessing associations between categorical variables, and the Wilcoxon rank-sum (Mann-Whitney) test was used for comparing medians of non-normally distributed continuous variables. A multivariate stepwise logistic regression was conducted to adjust for known cardiovascular risk factors and PCSK9 levels, aiming to identify their association with MALEs. To assess and minimize multicollinearity among covariates, we calculated the variance inflation factor (VIF) for all variables included in the multivariable model. Variables with critical collinearity were excluded, and all remaining variables exhibited acceptable VIF values (<2.5), ensuring the stability and robustness of the model. The effectiveness of PCSK9 levels in predicting MALEs was assessed by calculating the area under the receiver-operating characteristic (ROC) curve.

Additionally, a second predictive model was constructed that included PCSK9 levels and cardiovascular risk factors (such as age, sex, BMI, duration of diabetes, hypertension, hypercholesterolemia, history of CAD and CVD, smoking status, total cholesterol, LDL-C, triglycerides, fasting blood glucose, and HbA1c) to evaluate their collective predictive value for MALEs. The areas under the ROC curves of these models were compared using the Roccomp function in STATA software.

All statistical analyses were conducted using STATA version 18.0 for MacOS. A *p* -value of less than 0.05 was considered statistically significant.

## Results

### Characteristics of the Study Population

In this investigation, we examined 147 individuals with T2DM and PAD, all of whom required revascularization of the lower extremities for CLTI. The participants had a mean age (SD) of 75.2 (± 9.0) years. Among them, 99 (67.3%) were male and 48 (32.7%) were female. The mean duration of T2DM among the participants was 15 (4.25–30.0) years. Active smokers were 34 (23.1%), former smokers were 77 (52.4%), and patients who never smoked were 36 (24.5%). Hypertension was prevalent in 118 (80.3%) patients, while 135 (91.8%) suffered from hypercholesterolemia. A history of CAD was noted in 70 (47.6%) patients, and CVD history was present in 29 (20%). The severity of PAD was classified as Rutherford category 4 in 62 (42.2%) patients and category 5 in 85 (57.8%) patients. The average level of LDL-C was 68.0 mg/dL (49.0–86.0), and the mean HbA1c level was 6.9% (6.2–8.0). The mean circulating levels of PCSK9 stood at 379.1 ng/mL (± 105.6). Table 1 provides a detailed overview of the demographic and clinical characteristics of the study population.

**Table 1:** Demographic characteristics and clinical data of the study cohort at baseline

Number of patients	147
Men/female, *n*	99:48
Age, years ± SD	75.2 ± 9.0
Diabetes duration, years (IQR)	15 (4.25–30.0)
BMI, kg/m ^2^ (IQR)	25.6 (23.2–28.6)
Smoking (current), *n* (%)	34 (23.1)
Smoking (former), *n* (%)	77 (52.4)
Never smoked, *n* (%)	36 (24.5)
Hypertension, *n* (%)	118 (80.3)
Hypercholesterolemia, *n* (%)	135 (91.8)
CAD, *n* (%)	70 (47.6)
CVD, *n* (%)	29 (20.0)
Insulin, *n* (%)	64 (43.5)
Oral antidiabetics, *n* (%)	70 (47.6)
Statins, *n* (%)	105 (71.4)
Ezetimibe, *n* (%)	48 (32.7)
ACEi/ARB, *n* (%)	86 (58.5)
Other antihypertensive, *n* (%)	68 (46.3)
Aspirin, *n* (%)	85 (57.8)
Clopidogrel, *n* (%)	44 (29.9)
Low-dose rivaroxaban, *n* (%)	1 (0.7)
ABI (IQR)	0.39 (0.33–0.45)
Rutherford II-4, *n* (%)	62 (42.2)
Rutherford III-5, *n* (%)	85 (57.8)
Stenting, *n* (%)	3 (2.0)
HbA1c, % (IQR)	6.9 (6.2–8.0)
FBG, mg/dL (IQR)	120.5 (96.7–150.0)
Total cholesterol, mg/dL (IQR)	127.5 (109.5–152.0)
LDL cholesterol, mg/dL (IQR)	68.0 (49.0–86.0)
Non-HDL cholesterol, mg/dL (IQR)	90.0 (71.0–111.0)
Triglycerides, mg/dL (IQR)	105.0 (81.0–136.5)
Creatinine, mg/dL (IQR)	1.0 (0.8–1.5)
eGFR, mL/min/1.73 m ^2^ (IQR)	85.8 (68.9–93.7)
PCSK9, ng/mL ± SD	379.1 ± 105.6

Abbreviations: ABI, Ankle Brachial Index; ACEi, Angiotensin-Converting Enzyme inhibitor; ARB, Angiotensin II Receptor Blocker; BMI, body mass index; CAD, coronary artery disease; CVD, cerebrovascular disease; eGFR, estimated glomerular filtration rate; FBG, fasting blood glucose; HbA1c, glycated hemoglobin; PCSK9, proprotein convertase subtilisin/kexin type 9.

Note: The data are reported as the means ± standard deviations or median (interquartile range, IQR, 25–75) for continuous variables and as numbers (percentages) for categorical variables.

### Circulating PCSK9 Levels and Incidence of MALEs during the Follow-up Period after Lower-extremity Revascularization

During the 12-month follow-up period, 53 patients had a MALE after lower-extremity revascularization. No differences were observed among patients with and without MALEs regarding sex ( *p* = 0.40), diabetes duration ( *p* = 0.10), BMI ( *p* = 0.77), active smoking habit ( *p* = 0.76), history of high blood pressure ( *p* = 0.27), hypercholesterolemia ( *p* = 0.14), history of CAD ( *p* = 0.54), history of CVD ( *p* = 0.12), LDL-C levels ( *p* = 0.23), ABI ( *p* = 0.34), and eGFR ( *p* = 0.35). No significant differences were observed in the use of insulin, oral antidiabetic drugs, statins, ezetimibe, ACE inhibitors/ARBs, or antithrombotic therapies (aspirin, clopidogrel, low-dose rivaroxaban) between patients with and without MALEs.

Interestingly, patients with MALEs were younger than patients without MALEs ( *p <* 0.01). Moreover, patients with MALEs had a more severe PAD disease, presenting in the 74% of case with Rutherford 5 category ( *p* < 0.01) and higher levels of HbA1c (6.8 [6.2–7.8] vs. 7.5 [6.5–8.5], *p* < 0.01).

**Fig. 1:**
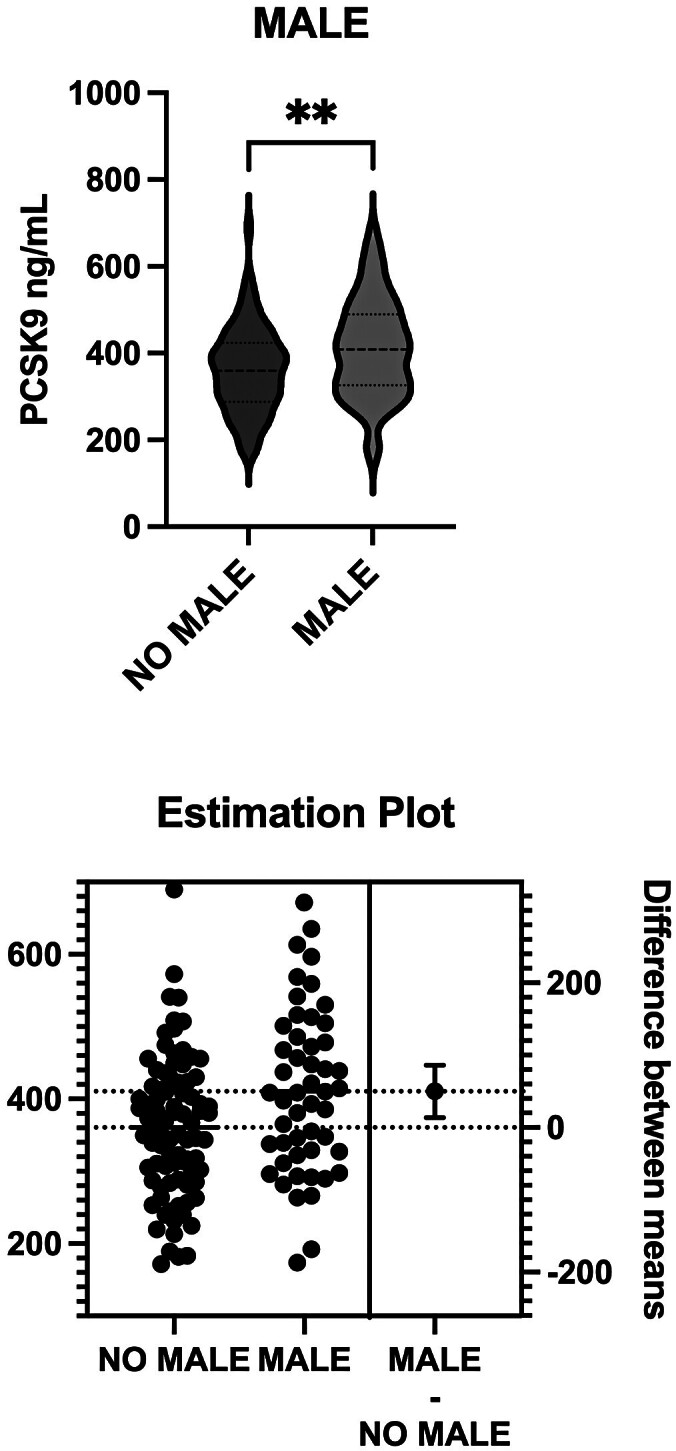
Circulating PCSK9 levels in patients with and without major adverse limb events (MALEs). The violin plot displays the distribution, median, and interquartile range (IQR) of serum PCSK9 concentrations in patients stratified by MALE status. Patients with MALEs showed significantly higher PCSK9 levels compared with those without MALEs (** indicates *p* < 0.01). The plot also illustrates the density of values within each group, providing a visual estimate of the distribution pattern.

Circulating PCSK9 levels were higher in patients with MALEs compared with those without MALE (410.5 ± 112.7 ng/mL vs. 360.6 ± 97.2 ng/mL, *p* < 0.01; Fig. 1 ). A violin plot was used to visualize the distribution and density of PCSK9 levels across groups, with clear separation between the two. The complete characteristics of the population with and without MALEs are described in Table 2 .

**Table 2:** Demographic and clinical data of study participants without or with MALE

	No MALE ( *n* = 94)	With MALE ( *n* = 53)	*p* -value
Men/female, *n*	61:33	38:15	0.40
Age, years ± SD	76.7 ± 8.6	72.6 ± 9.2	<0.01
Diabetes duration, years (IQR)	14.0 (2.0–25.0)	20.0 (7.2–30.0)	0.10
BMI, kg/m ^2^ (IQR)	25.6 (23.4–28.7)	24.9 (23.1–28.4)	0.77
Smoking (current), *n* (%)	21 (22)	13 (25)	0.76
Smoking (former), *n* (%)	47 (50.0)	30 (57)	0.44
Never smoked, *n* (%)	26 (28)	10 (19)	0.23
Hypertension, *n* (%)	78 (83.0)	40 (75)	0.27
Hypercholesterolemia, *n* (%)	84 (89)	51 (96)	0.14
CAD, *n* (%)	43 (46)	27 (51)	0.54
CVD, *n* (%)	15 (16)	14 (27)	0.12
Insulin, *n* (%)	26 (49.1)	38 (40.4)	0.40
Oral antidiabetics, *n* (%)	49 (52.7)	21 (39.6)	0.18
Statins, *n* (%)	40 (75.5)	65 (69.1)	0.53
Ezetimibe, *n* (%)	21 (39.6)	27 (28.7)	0.24
ACEi/ARB, *n* (%)	32 (60.4)	54 (57.4)	0.86
Other antihypertensive, *n* (%)	42 (44.7)	26 (49.1)	0.73
Aspirin, *n* (%)	34 (64.2)	51 (54.3)	0.32
Clopidogrel, *n* (%)	19 (35.8)	25 (26.6)	0.32
Low-dose rivaroxaban, *n* (%)	0 (0)	1 (1.1)	1.00
ABI, (IQR)	0.4 (0.33–0.45)	0.38 (0.33–0.45	0.34
Rutherford II-4, *n* (%)	48 (51)	14 (26)	<0.01
Rutherford III-5, *n* (%)	46 (49)	39 (74)	<0.01
Stenting, *n* (%)	3 (3.2)	0 (0)	0.55
HbA1c, % (IQR)	6.8 (6.2–7.8)	7.5 (6.5–8.5)	<0.01
FBG, mg/dL (IQR)	120.0 (95.0–146.0)	123.0 (100.5–159.0)	0.18
Total cholesterol, mg/dL (IQR)	130.5 (109.5–157.5)	125.5 (109.5–147.2)	0.47
LDL cholesterol, mg/dL (IQR)	70.0 (50.5–89.0)	60.0 (45.5–78.0)	0.23
Non-HDL cholesterol, mg/dL (IQR)	94.0 (70.0–115.0)	86.0 (71.5–98.0)	0.34
Triglycerides, mg/dL (IQR)	103.5 (76.5–139.5)	107.0 (91.0–132.0)	0.47
Creatinine, mg/dL (IQR)	1.0 (0.8–1.5)	1.0 (0.9–1.8)	0.18
eGFR, mL/min/1.73 m ^2^ (IQR)	86.2 (73.6–94.0)	84.3 (64.1–92.9)	0.35
PCSK9, ng/mL ± SD	360.6 ± 97.2	410.5 ± 112.7	<0.01

Abbreviations: ABI, Ankle Brachial Index; MALE, major adverse limb event; BMI, body mass index; CAD, coronary artery disease; CVD, cerebrovascular disease; ACEi, Angiotensin-Converting Enzyme inhibitor; ARB, Angiotensin II Receptor Blocker; eGFR, estimated glomerular filtration rate; FBG, fasting blood glucose; HbA1c, glycated hemoglobin; PCSK9, proprotein convertase subtilisin/kexin type 9.

Note: The data are reported as the means ± standard deviations or median (interquartile range 25–75) for continuous variables and as numbers (percentages) for categorical variables. Statistical tests were performed with Student's *t* -test, chi-square test or Wilcoxon rank-sum (Mann-Whitney) test, when appropriate.

**Fig. 2:**
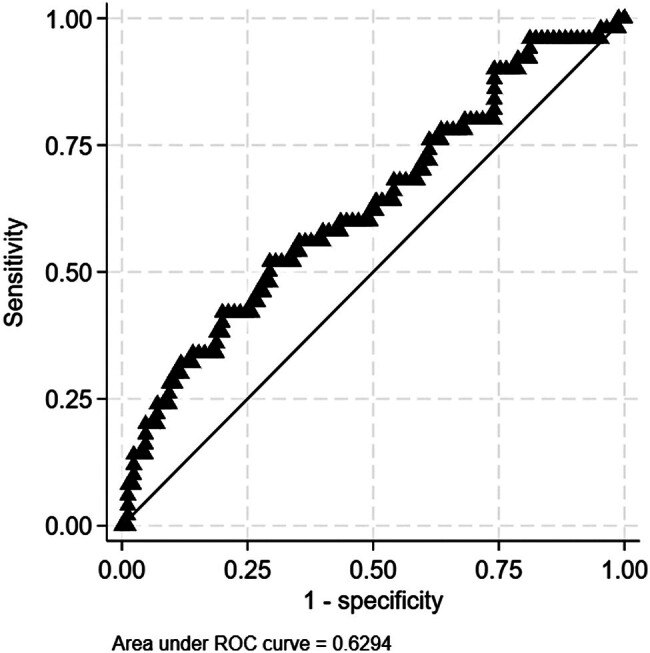
Receiver operating characteristic (ROC) curve for the prediction of major adverse limb events (MALEs) based on circulating PCSK9 levels. The true-positive rate (sensitivity) is plotted as a function of the false-positive rate (1 - specificity). The area under the curve (AUC) is 0.629.

**Fig. 3:**
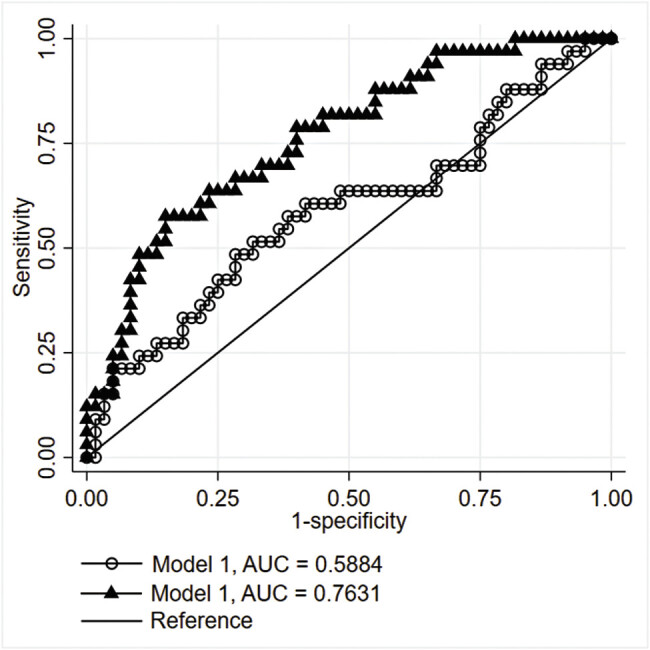
Comparison of receiver operating characteristic (ROC) curves for the prediction of major adverse limb events (MALEs). The true-positive rate (sensitivity) is plotted as a function of the false-positive rate (1 - specificity). The baseline model including traditional cardiovascular risk factors shows an area under the curve (AUC) of 0.589. The addition of circulating PCSK9 levels significantly improved the model's discriminative ability (AUC = 0.763, *p* < 0.05).

An ROC curve was elaborated to predict the incidence of MALEs based on circulating PCSK9 levels. The analysis yielded an area under the curve (AUC) of 0.629 (95% CI 0.530, 0.728), indicating modest but statistically meaningful discriminatory power in identifying patients at higher risk of adverse limb events ( Fig. 2 ). A second model was elaborated to predict the incidence of MALEs based on serum-circulating PCSK9 levels and cardiovascular risk factors (age, sex, diabetes duration, BMI, smoking status, hypertension, previous CAD and CVD history, total cholesterol, LDL-C, triglycerides, FBG, HbA1c). The comparison of ROC curves between the baseline model with only cardiovascular risk factors and the second model including circulating PCSK9 levels and cardiovascular risk factors demonstrated that including PCSK9 significantly improved ( *p* < 0.05) the prediction of MALEs, with an area under the ROC curve of 0.5889 (95% CI 0.461, 0.715) for the baseline model and 0.7631 (95% CI 0.662, 0.863) for the model including PCSK9 ( Fig. 3 ). This result highlights the incremental value of PCSK9 in risk stratification beyond established clinical variables.

After adjustment for traditional cardiovascular risk factors, a multivariable analysis was elaborated, showing that Rutherford 4 category ( *p* < 0.01) and circulating PCSK9 levels ( *p* < 0.05) were independent risk factors for MALEs in patients with PAD and CLTI requiring lower-extremity revascularization. The results are shown in Table 3 which reports the regression coefficients, standard errors, *p* -values, and 95% confidence intervals for each variable included.

**Table 3:** Multivariable logistic regression for MALE

MALE	Coef.	St.Err.	t-value	*p* -value	[95% confidence	interval]	Sig
Age	−0.01	0.01	−1.66	0.1	−0.02	0	
Male sex	0.04	0.11	0.34	0.73	−0.19	0.26	
BMI	0	0.01	−0.19	0.85	−0.02	0.02	
Diabetes duration	0	0	0.97	0.33	0	0.01	
Hypertension	−0.22	0.12	−1.87	0.06	−0.46	0.01	
Hypercholesterolemia	0.12	0.18	0.66	0.51	−0.24	0.47	
CAD	−0.09	0.1	−0.89	0.38	−0.3	0.11	
CVD	0.19	0.13	1.50	0.14	−0.06	0.45	
Smoking (current)	0.15	0.15	1.01	0.31	−0.14	0.44	
Smoking (former)	0.19	0.14	1.39	0.17	−0.08	0.46	
ABI	−0.3	0.71	−0.42	0.67	−1.71	1.11	
Rutherford II-4	−0.26	0.1	−2.66	0.01	−0.46	−0.07	**
LDL cholesterol	0	0	−1.01	0.31	−0.01	0	
FBG	0	0	0.03	0.98	0	0	
HbA1c	0.06	0.05	1.19	0.24	−0.04	0.17	
Creatinine	−0.02	0.04	−0.48	0.64	−0.11	0.07	
eGFR	−0.01	0	−1.85	0.07	−0.01	0	
PCSK9	0	0	2.04	0.04	0	0	*
Constant	1.02	0.83	1.22	0.23	−0.64	2.68	
Mean dependent var	0.36		SD dependent var		0.48		
R-squared	0.32		Number of obs.		104		
F-test	2.23		Prob > F		0.01		
Akaike crit. (AIC)	139.77		Bayesian crit. (BIC)		190.01		

Abbreviations: ABI, Ankle Brachial Index; BMI, body mass index; CAD, coronary artery disease; CVD, cerebrovascular disease; eGFR, estimated glomerular filtration rate; FBG, fasting blood glucose; HbA1c, glycated hemoglobin; MALE, major adverse limb event; PCSK9, proprotein convertase subtilisin/kexin type 9.

Notes: Multivariable logistic regression analysis for the prediction of major adverse limb events (MALEs).

The table reports regression coefficients (Coef.), standard errors (St.Err.), *p* -values, and 95% confidence intervals (CI) for each variable included in the model. Variables with statistically significant associations are marked (* *p* < 0.05; ** *p* < 0.01).

In a subgroup analysis limited to patients with Rutherford category 5 ( *n* = 85), circulating PCSK9 levels remained significantly associated with the occurrence of MALEs in a multivariate logistic regression model adjusted for major cardiovascular risk factors ( *p* = 0.03) ( Table 4 ).

**Table 4:** Multivariable logistic regression for MALE in Rutherford III-5 patients

	Coef.	St.Err.	t-value	*p* -value	[95% confidence	interval]	Sig
Age	0.923	0.039	−1.90	0.057	0.85	1.003	
Male sex	1.259	1.158	0.25	0.802	0.208	7.634	
BMI	1.052	0.087	0.61	0.54	0.895	1.237	
Diabetes duration	1.011	0.029	0.36	0.717	0.954	1.07	
Hypertension	0.089	0.11	−1.96	0.051	0.008	1.006	
Hypercholesterolemia	3.213	4.451	0.84	0.4	0.213	48.548	
CAD	0.519	0.421	−0.81	0.418	0.106	2.544	
CVD	7.962	9.105	1.81	0.07	0.847	74.89	
Smoking (current)	2.515	2.843	0.82	0.415	0.274	23.066	
Smoking (former)	4.986	5.232	1.53	0.126	0.638	38.988	
ABI	0.091	0.532	−0.41	0.683	0	9140.36	
LDL cholesterol	1.001	0.014	0.06	0.951	0.974	1.028	
FBG	0.999	0.009	−0.12	0.907	0.982	1.017	
HbA1c	1.514	0.652	0.96	0.336	0.651	3.522	
Creatinine	1.083	0.31	0.28	0.782	0.617	1.898	
eGFR	0.972	0.018	−1.52	0.128	0.937	1.008	
PCSK9	1.01	0.005	2.19	0.029	1.001	1.019	*
Constant	1.564	9.77	0.07	0.943	0	324,947.9	
Mean dependent var	0.469		SD dependent var		0.503		
Pseudo r-squared	0.296		Number of obs.		64		
Chi-square	26.170		Prob > chi2		0.071		
Akaike crit. (AIC)	98.303		Bayesian crit. (BIC)		137.163		

Abbreviations: ABI, Ankle Brachial Index; BMI, body mass index; CAD, coronary artery disease; CVD, cerebrovascular disease; eGFR, estimated glomerular filtration rate; FBG, fasting blood glucose; HbA1c, glycated hemoglobin; MALE, major adverse limb event; PCSK9, proprotein convertase subtilisin/kexin type 9.

Notes: Multivariable logistic regression analysis for major adverse limb events (MALEs) in patients classified as Rutherford category III-5.

The table reports regression coefficients (Coef.), standard errors (St.Err.), *p* -values, and 95% confidence intervals (CI) for each variable included in the model. In this subgroup analysis, PCSK9 remained significantly associated with MALEs (* *p* < 0.05).

## Discussion

In patients with T2DM and PAD undergoing endovascular revascularization for CLTI, we found that higher baseline circulating PCSK9 levels were independently associated with the occurrence of MALEs during follow-up. PCSK9 levels were also higher in younger patients and correlated with disease severity, in line with previous studies demonstrating similar patterns, particularly in coronary disease. 25 The link between diabetes and PAD remains a major health issue due to their combined vascular burden. 31

We have already shown a relationship between the biomarker of inflammation and risk of adverse cardiovascular outcomes in patients with T2DM and CLTI, who underwent lower-limb revascularization. 1 12 Interestingly, it has been demonstrated that elevated PCSK9 levels are associated with being a marker of subclinical atherosclerosis in patients with T2DM. 23 In addition, it was shown that increased PCSK9 levels corresponded to a significant increase in high-sensitivity C-reactive protein (hs-CRP), fibrinogen, and white blood cells (WBC), and a reduction in eGFR. 23

Although several previous evidence highlights a strong association between PCSK9 and cardiovascular risk, a correlation between circulating PCSK9 and adverse limb events after revascularization in PAD patients has not yet been evaluated. In our study, we found that the association between PCSK9 concentrations and MALEs persisted even after adjusting for traditional cardiovascular risk factors such as age, BMI, smoking habits, hypertension, blood lipids, glycemic control, and renal function, confirming PCSK9 as an independent risk factor for vascular events. In this context, we also evaluated non-HDL cholesterol levels, a broader marker of atherogenic burden. However, non-HDL cholesterol did not significantly differ between patients with and without MALEs, further supporting the hypothesis that conventional lipid markers may not fully account for residual limb risk in this population. Furthermore, in a subgroup analysis restricted to Rutherford category 5 patients, PCSK9 levels remained independently associated with MALEs after adjustment for major cardiovascular risk factors. This finding reinforces the role of PCSK9 as a robust predictor of adverse limb outcomes, even in patients with more advanced and homogeneous disease severity. In a large prospective cohort study, Leander et al demonstrated that higher baseline PCSK9 levels were independently associated with increased risk of various cardiovascular events, even in the absence of elevated LDL-C. 32 This finding is in line with our result. Indeed, in our cohort, there were no difference in terms of LDL-C levels among patients with and without MALEs.

The importance of PCSK9 levels as a key biomarker for diabetic vascular disease is supported by the findings of studies performed in individuals without diabetes. Ridker and colleagues in a nested case–control prospective study conducted in a cohort of healthy American women found that baseline circulating PCSK9 did not correlate with first cardiovascular events. 33 Studies in healthy subjects and patients with diabetes with unknown vascular disease are not entirely discordant with our study findings, or with findings of other multiple studies revealing the association of PCSK9 and cardiovascular disease. Findings should be interpreted as a plausible population-based evidence of the role of PCSK9 in diabetic patients as a determinant of vascular disease progression and as a biomarker of disease severity. Our study was conducted among patients with T2DM undergoing lower-extremity revascularization, representing a later stage in the natural progression of diabetic vascular disease. The hypothesis of temporal association between PCSK9 and vascular disease is further supported by findings of multiple observational and interventional studies establishing the role of PCSK9 inhibitors in reducing the risk of acute cardiovascular events in people with already known atherosclerotic cardiovascular disease (ASCVD). 19 20 21 Results from the classification analysis model in our study further support the hypothesis that PCSK9 biomarker level monitoring is particularly important in later stages of the peripheral vascular disease. ROC curve analysis showed that PCSK9 maintained its relevance as a predictive biomarker for MALEs even after adjustment for established cardiovascular risk factors. Although the discriminatory capacity of PCSK9 alone was limited, its clinical utility emerges when used in combination with traditional risk markers. In our cohort, adding PCSK9 to the multivariable model significantly improved its predictive accuracy, reinforcing the potential role of PCSK9 as a valuable adjunctive biomarker for risk stratification in T2DM patients with CLTI undergoing revascularization.

Multiple biological models support the predictive value of PCSK9 levels after revascularization as noted in the study, and the potential therapeutic role of PCSK9 inhibition to further reduce MALEs after revascularization in patients with diabetic vascular disease. PCSK9 is involved in inflammatory and thrombosis processes which could contribute to acute events after revascularization. 17 Recently, Shin and colleagues demonstrated that PCSK9 itself enhanced inflammation directly and independently from LDL metabolism, through a mechanism involving adenylyl cyclase-associated protein 1 (CAP1), spleen tyrosine kinase (Syk), and protein kinase C delta (PKCδ). Through the CAP1-Syk-PKCδ pathway, PCSK9 was able to induce NF-κB and inflammatory genes. 34 It increased the mRNA levels of pro-inflammatory cytokines and adhesion molecules, in particular TNF-α, IL − 1β, IL-6, VCAM1, ICAM1, and SELE, and scavenger receptors, like TLR-4 and LOX-1, 34 which are major mediators of LDL-C uptake by macrophages in the process of atherosclerosis. 17 Cheng et al found that higher circulating PCSK9 levels were associated with increased necrotic core content in coronary plaques, independent of LDL-C, supporting a direct role of PCSK9 in plaque inflammation and instability. 35 Another key biological pathway, further supporting the association of PCSK9 and MALEs after revascularization, is the effect of PCSK9 in platelet activation and aggregation, 36 enhancing thrombosis in a phenomenon that could contribute to acute adverse vascular events in patients with PAD undergoing revascularization. 10

Taken together, our findings provide crucial insights into the potential of PCSK9 as a biomarker for vascular complications, emphasizing the need for targeted management strategies in high-risk populations. The implications of the study are 2-fold. First, it underscores the importance of rigorous cardiovascular risk assessment and management in patients with T2DM and PAD, incorporating PCSK9 levels as part of the evaluation process. This approach could enhance the predictive accuracy for adverse outcomes, enabling clinicians to tailor interventions more effectively. Second, the findings suggest and advocate for the integration of PCSK9 inhibitors into the therapeutic arsenal for PAD in all stages of the disease, including patients undergoing revascularization. PCSK9 biomarker level monitoring should be considered as a therapeutic intervention beyond standard management of traditional cardiovascular risk factors.

The prospective design of our study and the rigorous assessment of PCSK9 levels and cardiovascular outcomes provide a robust framework for evaluating the biomarker's predictive value even in patients with an advanced phenotypic expression of diabetic vascular disease. Yet we acknowledge significant limitations. It was a single-center study with intrinsic confounding factors. A further limitation of our study is the absence of detailed procedural data, including lesion characteristics, type of devices used, final angiographic results, and skin perfusion pressure measurements. However, all patients underwent BTK revascularization using a standardized endovascular approach, and stent implantation was limited to only three patients (2.0%), which precluded any meaningful subgroup analysis. Although these data could have provided additional insights, the uniformity of the anatomical site and technique partially mitigates the potential impact of this limitation. In addition, a small sample size may limit generalizability of the results as compared with other population-based studies evaluating the role of PCSK9 as a biomarker at the population-based levels. However, our study is one of the largest prospective cohorts specifically designed to study the clinical and biomarker determinants of MALEs after revascularization, an understudied population exposed to an event thought to have distinctive and poorly understood pathophysiological characteristics that may lead to poor outcomes despite optimal medical care. In this context, the limitations of the study further highlight the importance of future research focusing on multicenter trials to validate the role of PCSK9 not only in patients with T2DM undergoing revascularization, but also across more demographically diverse populations and other types of health care settings.

In conclusion, our study highlights the significant association between PCSK9 levels and the risk of MALEs in patients with T2DM and PAD undergoing revascularization for CLTI. Findings of this study underscore the potential role of PCSK9 as a predictive biomarker and therapeutic target for personalized and effective management strategies beyond optimal management of traditional cardiovascular risk factors.

## Notes

**What is known about this topic?**

- In patients with diabetes, peripheral arterial disease (PAD) is linked to major adverse limb events (MALEs) and adverse cardiovascular outcomes.
- Patients with PAD have higher PCSK9 levels, and elevated levels correlate with an increased risk of PAD, independent of other risk factors.

**What does this paper add?**

- Findings highlight the potential role of PCSK9 as a predictive biomarker for assessing MALE risk.
- PCSK9 may serve as a therapeutic target for personalized and more effective management strategies.
